# Examining spatial microbiome variations across gastrointestinal tract regions in obesity

**DOI:** 10.1038/s41598-025-10931-0

**Published:** 2025-07-14

**Authors:** Jacqueline Rehner, Leidy-Alejandra G. Molano, Chara Christodoulou, Sebastian Holländer, Maximilian O. Förster, Verena Keller, Johannes Jäger, Sara Volz-Willems, Sören L. Becker, Matthias Glanemann, Michael Jelden

**Affiliations:** 1 Institute of Medical Microbiology and Hygiene, Saarland University, 66421 Homburg, Germany; 2 Chair for Clinical Bioinformatics, Saarland University, 66123 Saarbrücken, Germany; 3 Department of General Medicine, Saarland University, 66421 Homburg, Germany; 4 Clinic for General, Visceral, Vascular, and Thoracic Surgery, Klinikum Frankfurt Höchst, 65929 Frankfurt am Main, Germany; 5 Department of General, Visceral, Vascular and Paediatric Surgery, Saarland University, 66421 Homburg, Germany; 6 Department of Medicine II, Saarland University, 66421 Homburg, Germany; 7 Practice of General Medicine Dr. Jelden, 66421 Homburg, Germany

**Keywords:** Microbiome, Clinical microbiology

## Abstract

Obesity, a global health concern, is associated with alterations in the gut microbiome, yet its spatial dynamics across the gastrointestinal (GI) tract remain poorly understood. This study provides the first comprehensive analysis of the spatial microbiome in individuals with obesity, encompassing samples from the stomach, peritoneum, jejunum (50 cm and 150 cm post-Treitz), and feces before and after bariatric surgery. Using 16 S rRNA sequencing, we analyzed microbial composition and diversity after computational decontamination in 172 high-quality samples from 51 participants. Our results reveal significant differences in alpha diversity across GI sites, with stool samples exhibiting the highest diversity, which decreased post-surgery. The presence of microbial DNA in traditionally sterile compartments, such as the peritoneum and stomach, suggests potential microbial-host interactions warranting further exploration. Contaminants, including *Nitrotoga sp.*, were identified in low-biomass samples and excluded using computational decontamination, emphasizing the need for rigorous controls. This study establishes a foundation for spatial microbiome analysis in obesity, highlighting the impact of bariatric surgery and the importance of advanced multi-omics approaches to unravel host-microbiome dynamics.

**Supplementary Information:**

The online version contains supplementary material available at 10.1038/s41598-025-10931-0.

## Introduction

Obesity presents a pressing global health concern, marked by an excessive accumulation of especially visceral fat that elevates the risk for various diseases^1^. Within the intricate web of factors contributing to obesity, the human microbiome, particularly the gut microbiota, has emerged as a pivotal player in its pathophysiology^2–4^. Research has elucidated how the gut microbiome influences key metabolic pathways, regulates energy homeostasis, and modulates inflammatory processes, all of which significantly impact the onset and progression of obesity^5^.

In individuals with obesity, alterations in the composition and function of the gut microbiome have been observed, frequently manifesting as a decrease in microbial diversity and a shift in the relative abundance of specific bacterial taxa, such as an increase in *Bacillota* (*Firmicutes*) and a decrease in *Bacteroidota* (*Bacteroidetes*)^6^. Such dysbiosis within the gut microbiome can disrupt metabolic homeostasis, leading to increased energy harvest from the diet, enhanced adipogenesis, and systemic inflammation, all of which contribute to the development and progression of obesity^7–9^.

Moreover, the microbiome’s composition and activity are not static, but can be modulated by various factors, including diet, physical activity, medications, and disease states^10^. Dietary patterns rich in processed foods and high in fat and sugar have been associated with alterations in the gut microbiota, favoring the proliferation of pro-inflammatory bacteria and the depletion of beneficial microbes^10–12^. Conversely, diets high in fiber and plant-based foods promote the growth of health-promoting bacteria, fostering a more favorable microbial profile linked to metabolic health^10^.

Beyond diet, lifestyle factors such as physical activity and stress can also influence the gut microbiome composition and function^13^. Regular exercise has been shown to enhance microbial diversity and promote the growth of beneficial bacteria associated with metabolic health^13^. Conversely, chronic stress can disrupt the gut-brain axis, leading to alterations in gut permeability, immune function, and microbial composition, which may exacerbate metabolic dysfunction and obesity^13^.

Understanding the bidirectional relationship between the gut microbiome and obesity is crucial for developing targeted interventions aimed at modulating the microbiome to prevent or manage obesity and its associated metabolic complications. Despite significant strides in the gut microbiome’s involvement in obesity, the majority of studies have primarily relied on stool samples as a proxy for assessing gut microbial communities^14^. However, it is increasingly recognized that stool microbiota may not comprehensively represent the microbial populations inhabiting different segments of the gastrointestinal (GI) tract^15^. Recent technological advancements have paved the way for exploring the microbiome at various locations within the GI tract, offering a more nuanced understanding of its role in both health and disease^16^.

Considering this, our study introduces a novel approach by investigating the intraoperative microbiome of individuals suffering from obesity. By direct sampling from multiple sites, including the stomach and peritoneum, two distinct locations in the small intestine, and stool, our study provides a distinctive viewpoint on the spatial distribution of the microbiome within the GI tract concerning obesity. This methodological innovation enables a more precise characterization of the microbiome associated with obesity and sheds light on its potential implications for metabolic health.

## Methods

### Sampling

The study involved the collection of six samples from each participant. Four samples were obtained intraoperatively as follows:

1. Intraoperative: A swab was taken from the outside of the jejunum (peritoneum, in the collar pouch).
2. Intraoperative: A swab was collected from the interior of the jejunum at 50 cm from its opening.
3. Intraoperative: A swab was taken from the stomach.
4. Intraoperative: A swab was collected from the interior of the jejunum at 200 cm from its opening (50 cm biliopancreatic + 150 cm alimentary limb length) post-Treitz.

Sample collection was performed using provided swab tubes, specifically “DNA/RNA Shield Collection Tubes with Swabs” sourced from Zymo Research (Zymo Research Corp, Irvine, CA).

Additionally, two samples were obtained before and after surgery in the form of stool swabs:

5. Pre-operation: A stool sample was taken 1–2 days prior to surgery (e.g., on the day of admission).
6. Post-operation: A stool sample was collected on the 4th or 5th day following surgery.

A standardized minimally invasive Roux-en-Y gastric bypass was performed on all study participants. The biliopancreatic limb length was set at 50 cm, and the alimentary limb length at 150 cm, according to the standard in our clinic. Five trocar accesses were used in the upper abdomen: left lateral, right lateral, epigastric median, subcostal right, and subcostal left. A single shot antibiotic combination of ceftriaxone and metronidazole was given 30–60 min before beginning of surgery.

Immediately after creating the capnoperitoneum using a FIOS trocar (Applied Medical, California, USA) through an access in the left upper abdomen, the trocars were placed in the aforementioned standard positions. Following this, the peritoneal swab was taken in the Koller pouch. After creating the approximately 40 cc stomach pouch using endo-staplers (Endo GIA, Medtronic plc, Dublin, Ireland), a standard termino-lateral gastrojejunostomy was performed. First, the jejunal loop was opened with a diathermy hook 50 cm aboral to the Treitz ligament, and a swab was taken from the lumen of the opened loop. Subsequently, the stomach pouch was also opened with a diathermy hook, and another swab was taken from the gastric lumen. After completing the anastomosis, the foot-point anastomosis (jejuno-jejunostomy) was created 150 cm further aboral between the alimentary loop and the biliopancreatic loop using the same technique. After opening the alimentary loop, another swab was taken from its lumen.

All samples were frozen at – 80 °C immediately after the surgical procedure and stored for 12 months until further use. The samples were embedded in DNA/RNA Shield Collection Tubes provided by ZymoResearch to stabilize nucleic acids for up to 2 years.

All surgical procedures were performed by one experienced bariatric surgeon. The swabs were collected alternately through the working trocars to minimize the possibility of cross-contamination.

Three weeks prior to the surgery, patients were required to follow a crash-diet approach, consisting of the intake of three commercially available protein shakes of choice and a green salad in the evening. Up to six days after the operative procedure, patients received liquid foods only, consisting of broth and unsweetened and unflavoured yogurt.

### Microbial nucleic acid extraction

Metagenomic DNA extraction from fecal and intraoperative samples from the GI tract was conducted using the ZymoBIOMICS DNA Miniprep Kit (Zymo Research Corp, Irvine, CA), following the manufacturer’s protocol for isolation and purification. Initially, 50 mg of fecal matter underwent mechanical lysis using the MP Biomedicals™ FastPrep-24™ 5G Instrument (FisherScientific GmbH, Schwerte, Germany), with adjustments made to the manufacturer’s protocol regarding velocity and duration. Specifically, mechanical lysis was performed at 6 m/s for 45 s, repeated three times with 30 s intervals on ice between each lysis step. Swabs of the GI tract taken during surgery were vortexed rigorously to ensure the release of bacteria from the swab into the DNA/RNA shield medium provided by ZymoResearch. Then, 750 µl of medium, containing the microbes, was used for mechanical lysis as described above. To analyze potential contamination, we extracted DNA from the DNA/RNA Shield medium provided by ZymoResearch Corp., in which the samples were stored. For this, we used a fresh, unopened DNA/RNA Shield Collection Tube (ZymoResearch Corp., Irvine, CA) and treated the medium as described for all swab samples. To test the buffers used during DNA extraction (ZymoResearch Corp, Irvine, CA), we performed a DNA extraction with the ZymoBIOMICS DNA Miniprep Kit (ZymoResearch Corp, Irvine, CA) using 750 µl of sterile H_2_O. Subsequently, DNA was eluted in 70 µL of DNase-/RNase-free water. The concentration and purity of the eluted DNA was determined using NanoDrop 2000/2000c (ThermoFisher Scientific, Wilmington, NC, USA) through full-spectrum microvolume UV/Vis measurements^17^.

### 16 S rRNA sequencing

For profiling microbiome composition, the microbial DNA was sent to Novogene Company Limited (Cambridge, UK). To amplify the V3-V4 region of the 16 S ribosomal RNA gene, polymerase chain reaction (PCR) with the following primers was used: CCTAYGGGRBGCASCAG, GGACTACNNGGGTATCTAAT. Microbial samples and DNA-extraction blank controls were sequenced in separate PCR and sequencing runs. In total, 198 participant-derived samples were obtained and sequenced. However, due to insufficient DNA amounts, seven samples could not be sequenced and therefore had to be excluded from further analysis (P_056, M_038, M_056, M_069, J_2_012, S_1_073, and S_5_023).

### Statistical analysis of patients’ characteristics

Differences in clinical variables were assessed using the correspondent statistical test according to the characteristics of the variables. Chi-Square test was used for categorical variables, whereas t-test and non-parametric Mann-Whitney U-test were used for continuous variables. T-test was used for continuous variables with normal distribution and homogeneity of variances, whereas Welch correction was applied when heterogeneity of variances was detected. Mann-Whitney U-test was used for non-normal continuous data. P-values were corrected using Benjamini-Hochberg (BH) method.

### Amplicon sequencing analysis

16S ribosomal RNA (16S rRNA) raw sequence data was pre-processed using the QIIME2^18^ (version 2023.7) bioinformatics pipeline. The sequence reads were denoised, filtered out from chimeras, and de-replicated into amplicon sequence variants (ASVs) using DADA2^19^. Each ASV was taxonomically assigned to the species level using the QIIME2^20^ naive-bayes feature classifier with Greengenes2^21^ database as reference. The n-gram-range parameter was set to [7,7] and the confidence threshold to “disable” [PMID: 38189256]. Microbial samples were decontaminated using *decontam* package (v. 1.22.0) with the function: isContaminant(method=”combined”, neg=”is.neg”, conc=”Concentration_[ng/microl]”, threshold = 0.5)^22^. This combined method utilizes DNA concentration and negative controls to identify contaminants present in the samples, applying a threshold of 0.5^12,23^. Additionally, samples in which the proportion of contaminated species counts exceeded 79.33% (three times the standard deviation) were discarded from further analysis.

Differences in alpha diversity among groups were assessed using the non-parametric Wilcoxon signed-rank test (paired comparison) and p-values were adjusted with the ‘holm’ method. Alpha diversity indexes (Chao1, Shannon, and Simpson) were calculated using the vegan (v. 2.6-4) package.

Beta diversities were calculated using Bray-Curtis dissimilarities between samples and visualised using the Principal Component Analysis (PCoA) ordinations. The PERMANOVA test was used to analyze associations between microbial composition (beta diversity) and host factors (‘adonis2 permutation = 10000 by=“terms” seed = 123’).

The Analysis of Compositions of Microbiomes with Bias Correction 2 (ANCOM-BC2v.2.4.0)^23^ was used to assess differences in abundance between microbiome data and clinical variables (assay_name=”counts”, rand_formula = NULL, p_adj_method=”holm”, pseudo_sens = TRUE, prv_cut = 0.1, lib_cut = 0, s0_perc = 0.05, struct_zero = TRUE, neg_lb = TRUE).

The Spearman correlation test was used to compute correlations on cumulative sum scaled (CSS) counts. CSS transformation was applied using the cumNorm function from the metagenomeSeq package(v.1.43.0)^24^. Only correlation between the 20 most abundant species were computed.

## Results

### Patient characteristics

52 patients with a BMI between 30 and 50 were included in the study (BMI 33–36 *n* = 1; BMI 36–41 *n* = 4; BMI 41–46 *n* = 14; BMI 46–50 *n* = 16). Of the 52 patients, 72% were female and 28% were male. The age range varied from 24 to 70 years. After having obtained written informed consent, patients undergoing bariatric surgery at Saarland University Medical Center in the Clinic of General Surgery were included in the study from October 3, 2019, to December 12, 2022. Further inclusion criteria were the legal age of 18 years and the legal capacity to act. Exclusion criteria were lack of informed consent. Fatty liver, smoking and diabetic status, as well as CRP, IL-6 and HbA1c levels upon admission were evaluated (Table 1).

**Table 1:** Cohort characteristics.

Characteristic	*N*	Overall, *N*= 52	Gender		q-value*
			Female, *N*= 38	Male, *N*=14	
Age, Mean ± SD	51	47±11	47±11	47±13	>0.99
BMI, Mean ± SD	52	48±7	47±6	52±10	>0.99
CRP value at inclusion mgL, Mean ± SD	51	8±8	9±8	7±6	>0.99
IL-6 at inclusion pg-ml, Mean ± SD	47	5.57±3.23	5.41±3.03	6.05±3.84	>0.99
HbA1c perc, Mean ± SD	49	6.11±1.42	5.96±1.38	6.58±1.52	>0.99
Smoker, N%	52				>0.99
Yes		16 (31%)	11 (29%)	5 (36%)	
No		36 (69%)	27 (71%)	9 (64%)	
Fatty liver, N%	52				>0.99
Yes		38 (73%)	26 (68%)	12 (86%)	
No		14 (27%)	12 (32%)	2 (14%)	
Diabetes, N%	52				>0.99
Yes		24 (46%)	18 (47%)	6 (43%)	
No		28 (54%)	20 (53%)	8 (57%)	

The table shows the overall number of participants and participant characteristics such as the number of females and males, the BMI, CRP value, IL-6 value, HbA1c value, the existence of fatty liver and diabetes type 2, as well as the smoking behavior. Depicted are mean values and the respective standard deviation, as well as for disease and smoking behavior the percentages of participants.

### Microbial diversity and composition differ along the GI tract

A total of 52 patients with obesity that underwent bypass surgery were included in the study. Stool samples were obtained before and 4 to 5 days after the surgery, along with four different swabs during the bypass surgery: jejunum at positions 50 cm and 150 cm, peritoneum, and stomach. However, not all patients consented to the collection of all sample types, and seven specimens could not be successfully sequenced. Therefore, after sample collection, only 191 samples were considered for microbial data analysis (Supplementary Table 1).

**Fig. 1:**
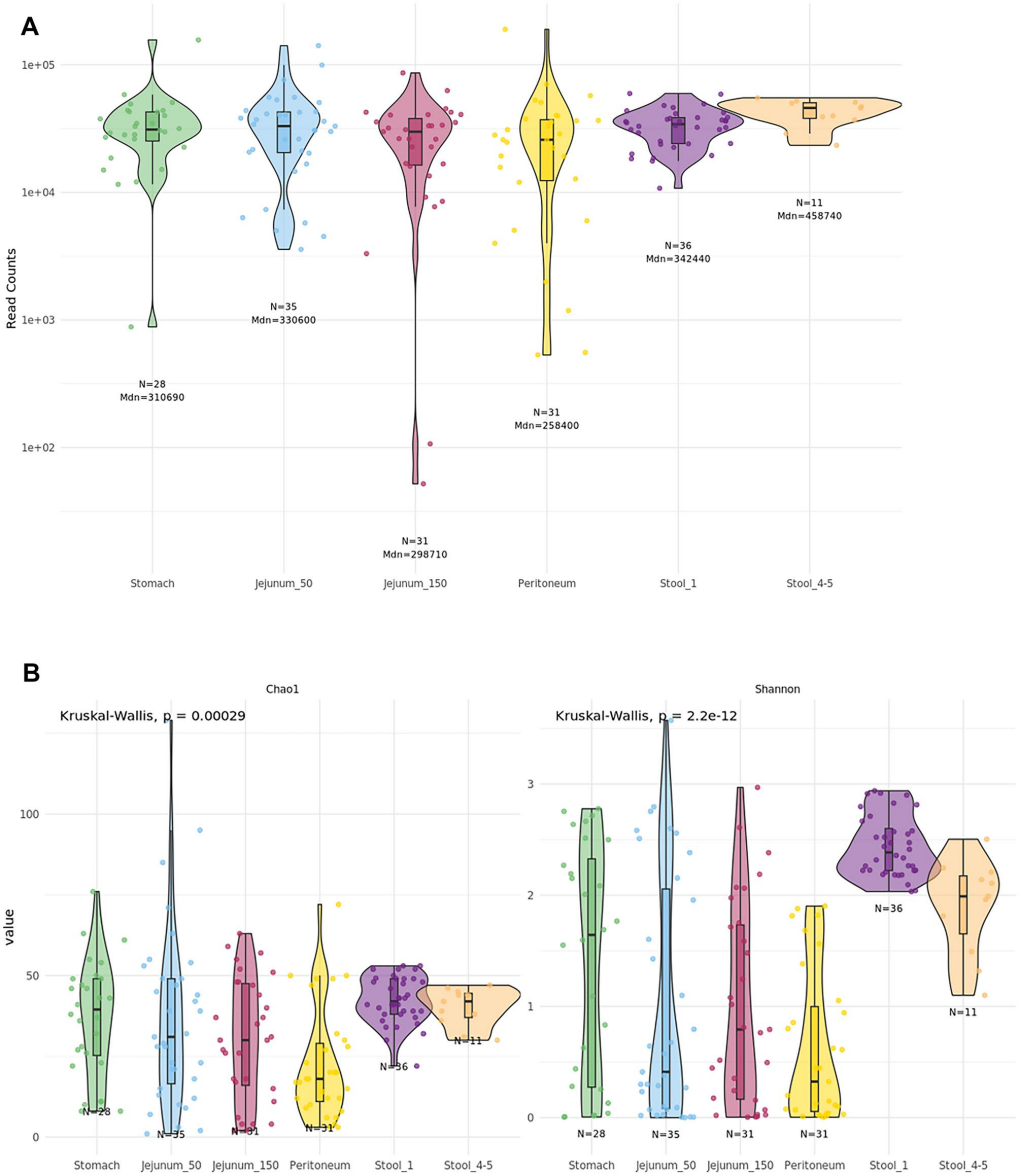
(**A**) Decontaminated species counts per sample type, coloured by sampling location. (**B**) Chao1 and Shannon alpha diversity indexes across the different sampling locations. Global differences between groups were assessed using the Kruskal-Wallis test and pairwise comparisons are specified in Table 2. *N* number of samples, *Mdn* median.

Microbial profiling of the swabs revealed that most of the stomach, jejunum, and peritoneum specimens were dominated by the presence of *Micrococcaceae* and *Nitrotoga sp003402285* (Supplementary Fig. 1). Due to the microbial low-biomass nature of these samples, we further wanted to assess if those microbes were contaminations either from the hands-on work during sampling or sample preparation, or pre-contamination of the medium used for the swabs. To evaluate this hypothesis, we performed nucleic acid extraction and sequencing of the storage medium contained in the swabs (Sample_ID: Puritan_I) and the buffers used during nucleic acid extraction (Sample_IDs: ZymoBuffer), provided by ZymoResearch. In Supplementary Fig. 2 the microbial profiling of the negative controls was represented. A total of 107 contaminants were detected during the decontamination analysis (Supplementary Fig. 2, Supplementary Table 2) and further removed from the samples (*see Methods*). Of note, whereas the suspected *Nitrotoga spp*. contaminant was detected, the second suspected contaminant belonging to *Micrococcaceae* was not detected as contaminant during the analysis. After decontamination, 172 samples from 51 patients were considered for subsequent analysis, yielding an average of 3.4 (sd +/- 1.77) sample types per subject (Supplementary Table 3). Each sample type yielded a median of 32,287.5 species counts (Fig. 1A), although stomach, jejunum, and peritoneum presented broader distributions than stool (Fig. 1A).

Furthermore, we assessed the alpha diversity across the different sample types (Fig. 1B; Table 2). Notably, the average alpha diversity of the peritoneum is similar to that of the jejunum, suggesting the presence of microbes or at least their genetic information in the peritoneal cavity. Additionally, the alpha diversity of stomach samples is comparable to that of both the peritoneum and jejunum. In contrast, stool samples collected before surgery exhibited significantly higher microbial diversity than other GI tract samples (p-value < 0.05). It is also noteworthy that microbial diversity in fecal samples decreased following surgery (Shannon, p-value = 0.00052). These effects could be due to the single shot antibiotics given before the surgery.

**Table 2:** Alpha diversity indexes comparisons across sampling locations.

Measure	Group1	Group2	*p*	*p*.adj	Method
Chao1	Peritoneum	Stool_1	2.30243E-06	0.000035	Wilcoxon
Chao1	Peritoneum	Stool_4-5	0.003500627	0.049	Wilcoxon
Chao1	Stomach	Peritoneum	0.004000271	0.052	Wilcoxon
Chao1	Jejunum_150	Stool_1	0.007222066	0.087	Wilcoxon
Chao1	Jejunum_50	Peritoneum	0.027937012	0.31	Wilcoxon
Chao1	Jejunum_50	Stool_1	0.049752229	0.5	Wilcoxon
Chao1	Jejunum_150	Peritoneum	0.108280779	0.97	Wilcoxon
Chao1	Jejunum_50	Jejunum_150	0.589354525	1	Wilcoxon
Chao1	Jejunum_50	Stomach	0.528965158	1	Wilcoxon
Chao1	Jejunum_50	Stool_4-5	0.327550343	1	Wilcoxon
Chao1	Jejunum_150	Stomach	0.210273158	1	Wilcoxon
Chao1	Jejunum_150	Stool_4-5	0.1606629	1	Wilcoxon
Chao1	Stomach	Stool_1	0.249541185	1	Wilcoxon
Chao1	Stomach	Stool_4-5	0.778700794	1	Wilcoxon
Chao1	Stool_1	Stool_4-5	0.339056869	1	Wilcoxon
Shannon	Peritoneum	Stool_1	1.6774E-19	2.5E-18	Wilcoxon
Shannon	Jejunum_150	Stool_1	9.05984E-11	1.3E-09	Wilcoxon
Shannon	Jejunum_50	Stool_1	5.68704E-07	0.0000074	Wilcoxon
Shannon	Peritoneum	Stool_4-5	3.01269E-06	0.000036	Wilcoxon
Shannon	Stomach	Stool_1	4.40728E-05	0.00048	Wilcoxon
Shannon	Stool_1	Stool_4-5	5.23746E-05	0.00052	Wilcoxon
Shannon	Jejunum_150	Stool_4-5	0.002862526	0.026	Wilcoxon
Shannon	Stomach	Peritoneum	0.004767997	0.038	Wilcoxon
Shannon	Jejunum_50	Stool_4-5	0.02187335	0.15	Wilcoxon
Shannon	Jejunum_150	Stomach	0.131683127	0.69	Wilcoxon
Shannon	Jejunum_150	Peritoneum	0.115307353	0.69	Wilcoxon
Shannon	Jejunum_50	Stomach	0.178729557	0.71	Wilcoxon
Shannon	Jejunum_50	Jejunum_150	0.749681532	0.92	Wilcoxon
Shannon	Jejunum_50	Peritoneum	0.306209263	0.92	Wilcoxon
Shannon	Stomach	Stool_4-5	0.396326961	0.92	Wilcoxon

p = p-value; p.adj = p-adjusted value.

**Fig. 2:**
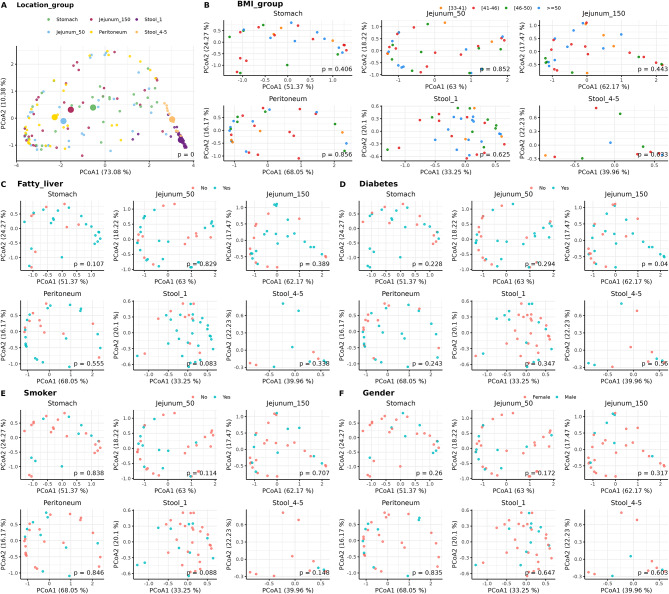
Principal component analysis (PCoA) ordinations showing the microbial composition of all samples (small dots), based on Bray-Curtis distances. Group centroids are represented by big dots. Differences and similarities based on (**A**) sampling location and traits within sampling locations: (**B**) BMI group, (**C**) fatty liver, (**D**) diabetes, (**E**) smoker status, and (**F**) gender. For each PCoA plot, PERMANOVA analysis was computed to assess significant differences between groups, with the p-value displayed. *p* p-value.

We compared the microbial composition of all samples using beta diversity measures, considering differences and similarities based on sampling location. Additionally, we examined factors such as BMI, the presence of fatty liver, smoking status, and gender to identify potential influences on microbiome variations across the GI tract (Fig. 2). The beta diversity analysis revealed no distinct cluster formation among intraoperatively collected GI tract samples. However, stool samples taken before and after surgery differed notably from each other and from samples obtained from the jejunum, peritoneum, and stomach (Fig. 2A). This effect might be due to the single dose antibiotic given immediately prior to surgery and the drastic change in diet after surgery (from a liquid diet consisting or protein shakes, and green salad followed by a transition to a wholesome diet after surgery). No cluster formation was observed for confounding factors such as BMI, diabetes, smoking habits, the presence of fatty liver, and biological sex (Fig. 2B-E).

**Fig. 3:**
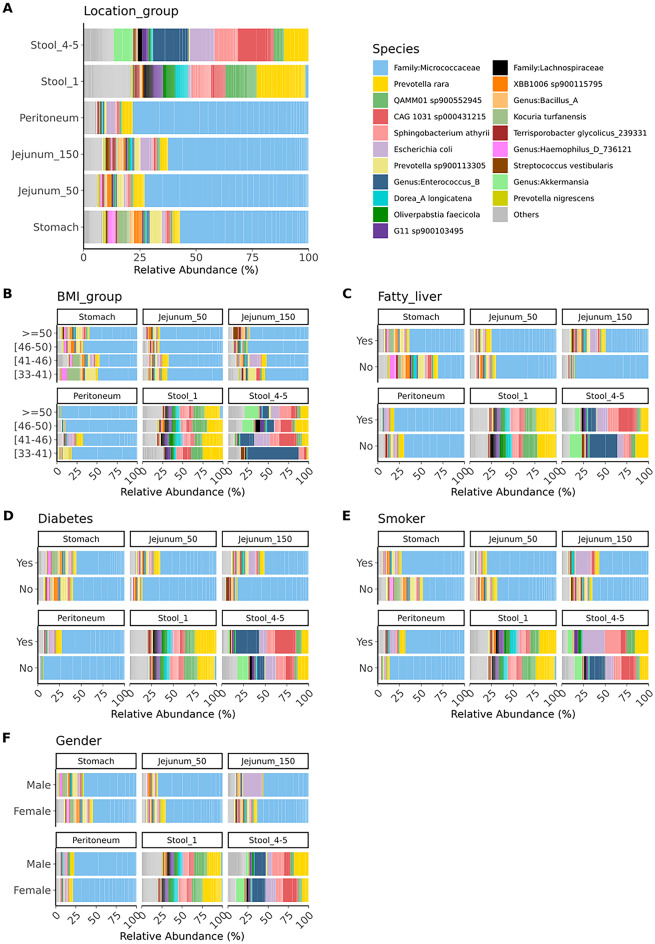
Relative abundance of microbes sorted according to (**A**) sampling location and traits within sampling locations: (**B**) BMI group, (**C**) fatty liver, (**D**) diabetes, (**E**) smoker status, and (**F**) gender. Labels are shown only for the most abundant taxa, collectively representing 90% of the total abundance. Low-abundance taxa are grouped under the ‘Others’ category.

To allow for a deeper insight into the microbial composition, we performed taxonomic profiling. Figure 3 shows the relative abundance of microbes sorted according to the sampling location (Fig. 3A), and confounding factors such as BMI group, the presence of diabetes or fatty liver, smoking status, and biological sex (Fig. 3B-F). In the jejunum at 150 cm, *E. coli* and *Streptococcus vestibularis* show high relative abundances. Stomach samples exhibit a similar relative abundance pattern, with *Haemophilus_D_736121* and *Prevotella sp900113305* being more abundant. In peritoneum samples, *Prevotella rara* and *E. coli* are the most abundant species. All intraoperatively collected samples show a high relative abundance (approximately 60–85%) of the *Micrococcaceae* family, which we could not identify as contamination. Stool samples taken before surgery contain higher levels of *QAMM01 sp900552945*, *Dorea_A longicatena*, and *Prevotella rara*, while after surgery, *Akkermansia*, *Enterococcus_B*, *E. coli*, and *CAG 103 sp000431215* are more abundant. In participants with a BMI of 50 and above, *S. vestibularis* is abundant in the jejunum (150 cm). In those with a BMI between 41 and 46, *E. coli* is prevalent in the jejunum (150 cm), and the peritoneum contains larger amounts of *E. coli* and *P. rara*. Stool samples after surgery in participants with a BMI between 33 and 41 are dominated by *Enterococcus_B*. In stomach samples, *Micrococcaceae* show a high relative abundance. Stool samples obtained after surgery contain higher levels of *Enterococcus_B*, while *CAG 1031 sp000431215* and *Sphingobacterium athyrii* are present in distinct proportions. In participants with diabetes, lower relative abundances of *Micrococcaceae* are observed in the GI tract. Stool samples after surgery contain larger amounts of *Enterococcus_B* and *CAG 1031 sp000431215*. Smoking influences bacterial composition, particularly in the jejunum (150 cm) and stool samples after surgery, where *E. coli* is highly abundant. In males, the jejunum (150 cm) also shows a high presence of *E. coli*. Most smokers with jejunum (150 cm) samples (8 out of 10) are female, indicating no pattern in the dataset.

### Spatial Microbiome analysis reveals fecal core microbes but highlights unique individual composition

**Fig. 4:**
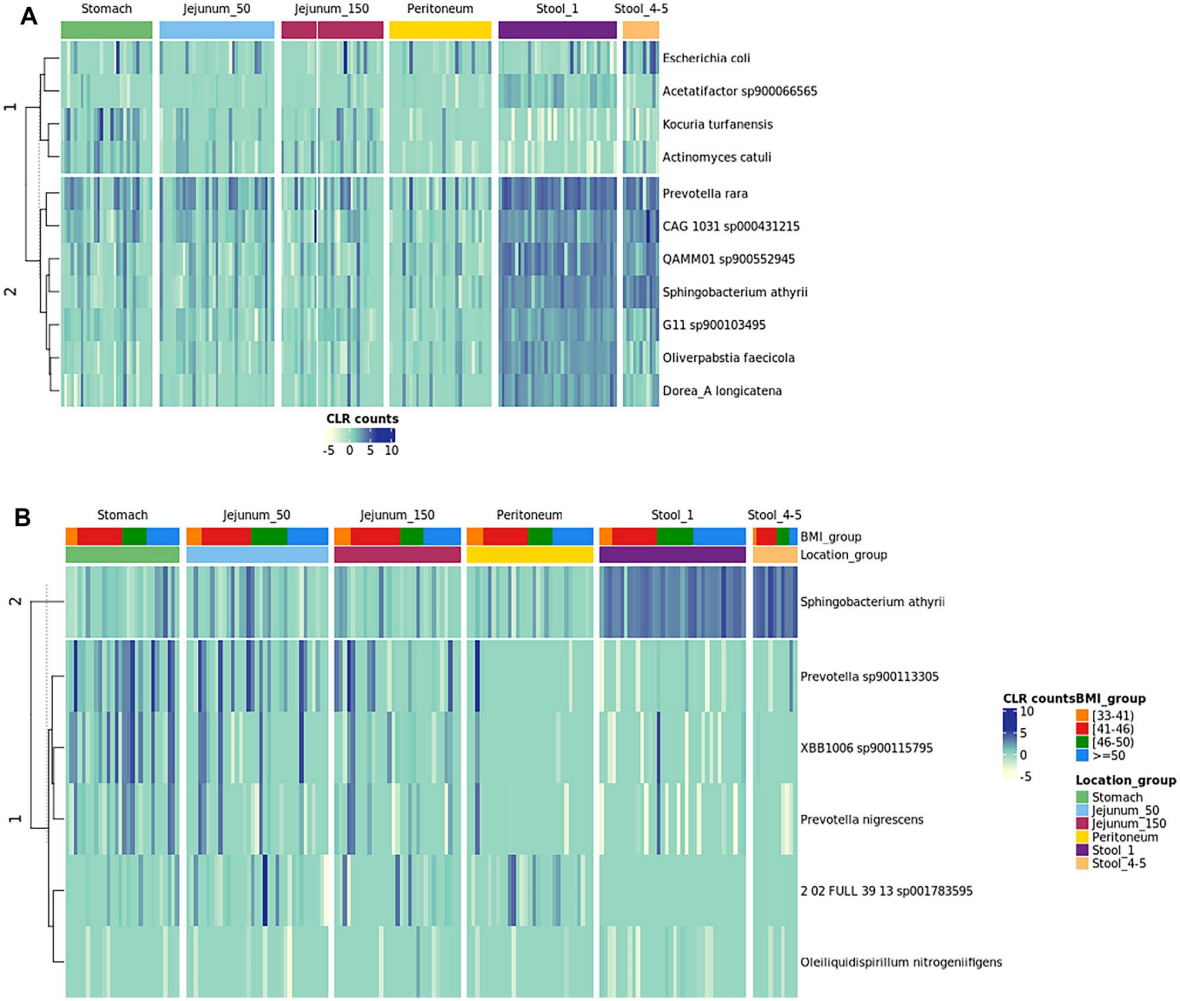
(**A**) Heatmap displaying centered log-ratio (CLR) transformed species counts for differentially abundant species across sampling locations (details on Table 3). Species were clustered into two groups based on k-means clustering. (**B**) Heatmap displaying centered log-ratio (CLR) transformed species counts for differentially abundant species across BMI groups within sampling locations (details on Table 4). Species were clustered into two groups based on k-means clustering.

**Fig. 5:**
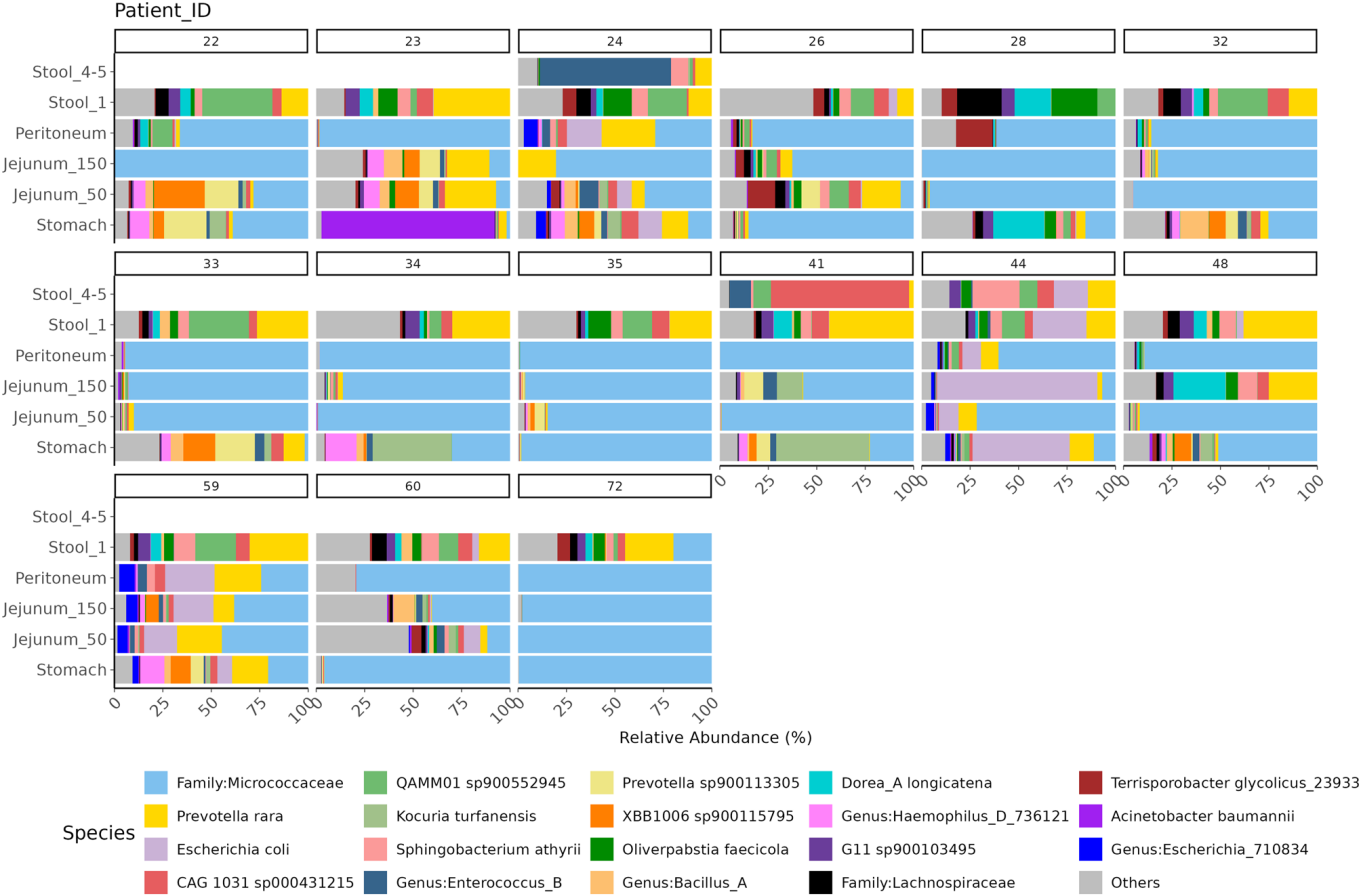
Relative abundance of individual microbiomes according to sampling locations. Species labels are shown only for the most abundant taxa, collectively representing 90% of the total abundance. Low-abundance taxa are grouped under the ‘Others’ category. Only individual microbiomes with samples available at least from the following locations are displayed: Stomach, Jejunum50, Jejunum150, Peritoneum, and Stool_1.

The microbial composition of 19 patients of which we obtained at least swabs from the stomach, jejunum 50 cm, jejunum 150 cm, peritoneum, and the pre-operative stool sample, were included in the analysis. As the microbiome is as unique as the human fingerprint, we also looked at individual microbiomes. Differential abundance analysis revealed 11 bacterial taxa that differ in abundance between the GI tract locations. No significantly differential abundant species were detected between the different jejunum samples, nor the stool samples taken before and after surgery. The most significant results were obtained for *Oliverpabstia faecicola* being more abundant in stool samples than in stomach samples (q-value = 7,6 × 10^−5^). Furthermore, *P. rara* was significantly more abundant in the peritoneum than in stool before surgery (q-value = 9,91 × 10^−5^) (Fig. 4a; Table 3). Differential abundance analysis was also performed for the different GI tract locations and BMI groups (Fig. 4b; Table 4). Six species were highlighted to be differentially abundant. *Prevotella nigrescens* (q-value = 0,00684), *Prevotella sp900113305* (q-value = 0,0355), and *Sphingobacterium athryrii* (q-value = 0,01895) were significantly more abundant in stomach samples from participants with a BMI of 50 or larger compared to those with a BMI between 41 and 46. In stool samples before surgery, *Oleiliquidispirillum nitrogeniifigens* (q-value = 0,04996) was significantly more abundant in samples from participants of the BMI group 46–50, compared to those with a BMI of 41–46. Notably, no significant differential abundant species were observed between the lowest and highest BMI group. Furthermore, stool samples taken after surgery change in their relative abundance in comparison to the stool samples taken prior. As visualized in Fig. 5, three stool samples were collected after surgery from patient 024, 041, and 044. For patient 024, the stool sample prior to surgery was very diverse in terms of relative abundance of bacteria, however after surgery it is dominated by *Enterococcus* B. Patient 041 for example was predominated by *Prevotella rara*, which then switched to mainly *CAG 1031 sp000431215* after the operative procedure (Fig. 5). Looking at peritoneum swabs, for most patients only *Micrococcaceae* are detectable, however for patient 022, 024, 028, 044, and 059 the microbial composition was more diverse. Similar trends hold true for the jejunum samples. For some patients, many bacteria can be identified, for others only *Micrococcaceae*. For swabs taken from the stomach, patient 023 shows mainly *Acinetobacter baumanii*, whereas others are either quite diverse or dominated by *Micrococcaceae* sp. For most fecal samples, we were able to detect e.g. *Sphingobacterium athyrii*,* Prevotella rara*,* CAG 1031 sp000431215*, and QAMM01 sp900552945, suggesting that these bacteria might contribute to the core composition of the colon (Fig. 5).

**Table 3:** Differential abundant species across sampling locations.

Taxon	Comparison_groups	*p*	q	lfc	passed_ss	W
Acetatifactor sp900066565	Stomach_vs_Stool_1	2.779E-03	3.89E-02	2.7117	TRUE	3.1296
Acetatifactor sp900066565	Stool_1_vs_Peritoneum	4.183E-04	6.27E-03	3.3735	TRUE	3.7521
Actinomyces catuli	Stomach_vs_Peritoneum	6.538E-05	9.81E-04	-3.5693	TRUE	-4.2515
Actinomyces catuli	Stomach_vs_Stool_1	8.563E-05	1.20E-03	-3.4826	TRUE	-4.1748
CAG 1031 sp000431215	Stomach_vs_Stool_1	2.312E-03	2.77E-02	2.2852	TRUE	3.1092
CAG 1031 sp000431215	Stool_1_vs_Jejunum_150	1.882E-03	2.45E-02	2.8887	TRUE	3.1743
CAG 1031 sp000431215	Stool_1_vs_Peritoneum	8.941E-05	1.34E-03	3.5629	TRUE	4.0464
CAG 1031 sp000431215	Stool_4-5_vs_Jejunum_150	3.181E-03	3.50E-02	3.5299	TRUE	3.0065
CAG 1031 sp000431215	Stool_4-5_vs_Peritoneum	3.916E-04	5.48E-03	4.2041	TRUE	3.6420
Dorea_A longicatena	Stomach_vs_Stool_1	1.323E-05	1.99E-04	3.8168	TRUE	4.6072
Dorea_A longicatena	Stool_1_vs_Jejunum_150	1.825E-03	2.19E-02	2.9265	TRUE	3.2116
Dorea_A longicatena	Stool_1_vs_Jejunum_50	2.425E-04	3.39E-03	3.2715	TRUE	3.8213
Dorea_A longicatena	Stool_1_vs_Peritoneum	1.342E-03	1.74E-02	2.9721	TRUE	3.3091
Escherichia coli	Stomach_vs_Stool_4-5	3.775E-03	4.91E-02	3.6742	TRUE	2.9997
Escherichia coli	Stool_4-5_vs_Jejunum_150	3.228E-03	4.52E-02	3.6261	TRUE	3.0534
Escherichia coli	Stool_4-5_vs_Jejunum_50	1.049E-03	1.57E-02	3.9025	TRUE	3.4241
G11 sp900103495	Stomach_vs_Stool_1	1.636E-03	2.13E-02	2.3551	TRUE	3.2278
G11 sp900103495	Stool_1_vs_Jejunum_150	3.668E-03	4.40E-02	2.6180	TRUE	2.9678
G11 sp900103495	Stool_1_vs_Jejunum_50	5.256E-04	7.36E-03	2.9613	TRUE	3.5709
G11 sp900103495	Stool_1_vs_Peritoneum	9.577E-05	1.44E-03	3.4991	TRUE	4.0469
Kocuria turfanensis	Stomach_vs_Peritoneum	3.305E-04	4.63E-03	-3.3068	TRUE	-3.7822
Kocuria turfanensis	Stomach_vs_Stool_1	1.284E-05	1.93E-04	-4.1011	TRUE	-4.7060
Kocuria turfanensis	Stool_1_vs_Jejunum_150	1.723E-03	2.24E-02	-3.0018	TRUE	-3.2637
Oliverpabstia faecicola	Stomach_vs_Stool_1	5.070E-06	7.60E-05	3.7106	TRUE	4.8124
Oliverpabstia faecicola	Stool_1_vs_Jejunum_150	3.080E-04	3.70E-03	3.3608	TRUE	3.7339
Oliverpabstia faecicola	Stool_1_vs_Jejunum_50	1.039E-04	1.35E-03	3.3774	TRUE	4.0365
Oliverpabstia faecicola	Stool_1_vs_Peritoneum	4.640E-05	6.50E-04	3.7503	TRUE	4.2519
Prevotella rara	Stomach_vs_Stool_1	2.498E-04	3.25E-03	2.7937	TRUE	3.7657
Prevotella rara	Stool_1_vs_Jejunum_150	2.176E-04	3.05E-03	3.4945	TRUE	3.8039
Prevotella rara	Stool_1_vs_Jejunum_50	3.453E-03	4.14E-02	2.5243	TRUE	2.9784
Prevotella rara	Stool_1_vs_Peritoneum	6.605E-06	9.91E-05	4.2811	TRUE	4.6960
Prevotella rara	Stool_4-5_vs_Peritoneum	3.784E-03	4.16E-02	3.5723	TRUE	2.9484
QAMM01 sp900552945	Stomach_vs_Stool_1	3.446E-05	5.17E-04	3.3467	TRUE	4.3081
QAMM01 sp900552945	Stool_1_vs_Jejunum_150	1.788E-04	2.15E-03	3.5561	TRUE	3.8713
QAMM01 sp900552945	Stool_1_vs_Jejunum_50	1.009E-04	1.31E-03	3.5118	TRUE	4.0266
QAMM01 sp900552945	Stool_1_vs_Peritoneum	3.598E-05	5.17E-04	3.8722	TRUE	4.2970
Sphingobacterium athyrii	Stomach_vs_Stool_1	9.744E-06	1.46E-04	3.5078	TRUE	4.6403
Sphingobacterium athyrii	Stomach_vs_Stool_4-5	2.071E-04	2.07E-03	4.1235	TRUE	3.8398
Sphingobacterium athyrii	Stool_1_vs_Jejunum_150	1.286E-04	1.67E-03	3.5332	TRUE	3.9711
Sphingobacterium athyrii	Stool_1_vs_Jejunum_50	1.536E-04	1.84E-03	3.3322	TRUE	3.9226
Sphingobacterium athyrii	Stool_1_vs_Peritoneum	4.484E-05	6.28E-04	3.7576	TRUE	4.2523
Sphingobacterium athyrii	Stool_4-5_vs_Jejunum_150	3.195E-04	2.88E-03	4.1488	TRUE	3.7175
Sphingobacterium athyrii	Stool_4-5_vs_Jejunum_50	4.436E-04	3.55E-03	3.9479	TRUE	3.6232
Sphingobacterium athyrii	Stool_4-5_vs_Peritoneum	1.542E-04	1.84E-03	4.3733	TRUE	3.9214

Only significant abundant taxa (q < 0.05) are displayed. All possible pairwise comparisons between compartments were performed. The comparison_groups column indicates the pairwise comparison where the taxon was found differentially abundant, taking the first compartment as reference. Abbreviations: lfc = log-fold change, W = test statistics, q = adjusted p-value, p = p-value, passed_ss = sensitivity analysis passed.

**Table 4:** Differential abundant species across BMI groups, within sampling locations.

taxon	Location_group	comparison_var	comparison_groups	*p*	q	lfc	passed_ss	W
Prevotella nigrescens	Stomach	BMI_group	[41-46)_vs_>=50	0.000760962	0.006848656	2.725139967	FALSE	4.473527568
Prevotella sp900113305	Stomach	BMI_group	[41-46)_vs_>=50	0.003945819	0.035512372	2.351625296	FALSE	3.495783904
Sphingobacterium athyrii	Stomach	BMI_group	>=50_vs_[46-50)	0.002105872	0.018952847	2.772928658	FALSE	3.824784908
Sphingobacterium athyrii	Stomach	BMI_group	[41-46)_vs_>=50	0.002370402	0.018963215	1.9865911	FALSE	3.762505126
XBB1006 sp900115795	Stomach	BMI_group	[41-46)_vs_[46-50)	0.004652798	0.04187518	2.630268376	FALSE	3.361867592
2 02 FULL 39 13 sp001783595	Jejunum_50	BMI_group	>=50_vs_[46-50)	0.000239504	0.006227103	-3.943112882	FALSE	-5.153729504
2 02 FULL 39 13 sp001783595	Jejunum_50	BMI_group	[41-46)_vs_[46-50)	0.000119455	0.003225286	3.695959261	FALSE	5.582312347
Oleiliquidispirillum nitrogeniifigens	Stool_1	BMI_group	[41-46)_vs_[46-50)	0.001921549	0.049960262	2.277415763	FALSE	4.324122612

Only significant abundant taxa (q < 0.05) are displayed. Abbreviations: lfc = log-fold change, W = test statistics, q = adjusted p-value, p = p-value, passed_ss = sensitivity analysis passed.

### Certain bacterial species seem to correlate with the presence of others

Next, we further investigated whether any bacterial species only occur in the presence of others, suggesting, for example, metabolic dependence between species (Supplementary Fig. 3, Supplementary Table 4). The highest correlation observed is between *Lachnospiraceae* and *G11 sp900103495* (Spearman correlation = 0.852, p-value = 0), suggesting a potential interaction between the two. This could, for example, be due to metabolic products of one species which nurture the other, or milieu changes that enhance growth of the other species. Further, *Dorea_A longicatena* and *Oliverpabstia faecicola* correlated in their presence with a Spearman correlation of 0.85 (q-value = 0). No Spearman correlation values above 0.852 were observed, suggesting that species can also survive in the respective compartment of the GI tract without the presence of another.

## Discussion

This study represents, to our knowledge, the first spatial microbiome analysis of the GI tract in humans with obesity, encompassing samples from the stomach, peritoneum, and various locations within the jejunum. Previous investigations into the microbial composition of the jejunum have primarily utilized endoscopic procedures or specialized catheters, which may be contaminated by oral, esophageal, stomach, and duodenal microbes before reaching the jejunum^25,26^. Additionally, microbiome analyses in the context of obesity have largely relied on human-derived fecal samples or murine models^8^. Notably, the microbiomes of the peritoneum and stomach in patients with obesity have not been previously characterized.

Our study revealed the presence of microbial DNA in the peritoneal cavity, suggesting potential microbial-host interactions and signaling within this compartment. While these findings do not confirm the presence of living bacteria in the peritoneum, the detection of microbial nucleic acids indicates its significance in gut microbial signaling, a concept supported by studies on diabetic mice^27,28^. These observations warrant further investigation into the role of the peritoneal microbiome in metabolic diseases such as type 2 diabetes and obesity. Future research should incorporate microbiological culturing and metabolomics to determine whether viable bacteria or merely their metabolites and nucleic acid residues exist in the peritoneum without inducing inflammation.

Similarly, we detected bacterial nucleic acids in the stomach, an environment traditionally considered hostile to microbial survival, except for some species that withstand the acidic conditions, such as *Helicobacter pylori*, which is commonly found in the gastric environment^29^. The detection of microbial DNA in stomach samples underscores the need for future studies to culture stomach swabs and explore the potential health implications of bacteria and archaea capable of thriving in acidic conditions.

Our analysis of fecal samples collected before and four to five days after surgery demonstrated a significant reduction in alpha diversity postoperatively. This decrease may be attributed to the surgical procedure itself or the consequent reduced caloric and nutrient intake following at least six days post gastric bypass surgery. Longitudinal studies are required to determine whether microbial diversity returns to baseline levels over extended periods and if this correlates with patient weight loss. We also observed postoperative shifts in microbial composition, with a non-significant increase in *Enterococcus B*, *Akkermansia sp*., and *Escherichia coli*, and a non-significant decrease in *Oliverpabstia faecicola*, *Dorea longicatena*, and QAMM01 sp900552945. Similar microbial shifts have been reported in patients with obesity six months post-gastric bypass surgery^30^with compositional stability observed at 12 months postoperatively^31^. As these results only highlight trends and not significantly differentially abundant bacterial species prior and post-surgery, we would suggest to increase the sample size in following studies to ensure if these trends entail significant changes that should be investigated in more detail.

In examining the jejunum, we investigated potential differences in bacterial composition at depths of 50 cm and 150 cm. Our findings indicated similar taxa at both sampling sites, with variations in relative abundances. These results align with Villmones et al. (2022), who reported no core microbiota in the jejunum but rather significant interindividual variability^32^. They identified *Streptococcus mitis*, *Streptococcus sanguinis*, and *Gemella haemolysans*, typically oral cavity inhabitants, along with *Granulicatella adiacens* and *Schaalia odontolytica*, in the GI tract. Our cohort similarly exhibited high relative abundances of *Prevotella rara* in the jejunum, followed by *E. coli*, and other *Prevotella species*.

Due to the detection of *Micrococcaceae* in all swab samples, we tried to identify a potential contamination. However, in silico decontamination analysis did not reveal *Micrococcaceae* as a contaminant. However, studies suggest the presence of different *Micrococcus* species in the air of medical environments, which could have been the case in our setting as well^33–36^.

Our study’s limitations include the exclusive use of 16 S rRNA sequencing, which due to the use of PCR before might miss specific bacterial nucleic acids where the primers do not align well, and overrepresent bacteria that are more abundant in the sample whereas low abundancies are overseen. Furthermore, by using 16 S rRNA sequencing, we only receive information on the taxonomic level, however no information on the functional capacity of the microbes. Future research should adopt an omics approach, combining metagenomics and metabolomics to elucidate bacterial metabolites and their genetic capacities in relation to host health. Detailed environmental characterization of sample sites, such as pH levels, is also crucial as these factors significantly influence microbial composition. Long-term studies are needed to assess the stability of the colonic microbiome post-surgery and its correlation with weight loss and health improvements. Nevertheless, this study is pioneering in describing the spatial microbiota composition across various GI tract sites within the same patient cohort, unimpeded by sampling method limitations. This comprehensive approach moves beyond fecal samples as proxies, including analyses of distinct jejunal sites and traditionally sterile compartments such as the peritoneum and stomach. The viability and metabolic activity of detected microbes, however, remain to be determined.

## Conclusion

This study provides the first comprehensive spatial microbiome analysis of the gastrointestinal tract in individuals with obesity, including the stomach, peritoneum, different jejunal locations, and feces as a proxy for the colon. Additionally, to our knowledge, this is the first study to examine the microbiome of the peritoneum in humans. Our findings highlight the presence of microbial DNA in traditionally sterile compartments like the peritoneum and stomach, suggesting potential microbial-host interactions that merit further investigation, particularly in the context of metabolic diseases such as type 2 diabetes and obesity. Postoperative reductions in microbial diversity and shifts in composition underscore the significant impact of gastric bypass surgery on the gut microbiome. While our study underscores the necessity of advanced omics approaches and detailed environmental characterization for future research, it establishes a foundational understanding of GI tract microbiota in obesity, moving beyond fecal samples to provide a more holistic assessment. Further studies are essential to explore the viability and metabolic activity of detected microbes and their implications for health and disease.

## Electronic supplementary material

Below is the link to the electronic supplementary material.

Supplementary Material 1

Supplementary Material 2

Supplementary Material 3

Supplementary Material 4

Supplementary Material 5

Supplementary Material 6

Supplementary Material 7

Supplementary Material 8

Supplementary Material 9

## Data Availability

The 16 S rRNA sequencing data is available under the accession number PRJNA1227268 on the BioProject database.
