# Supplementary figures and images for: Examining spatial microbiome variations across gastrointestinal tract regions in obesity
Source: Sci Rep. 2025 Jul 14;15:25423. doi: 10.1038/s41598-025-10931-0 (PMC12260093; doi:10.1038/s41598-025-10931-0)

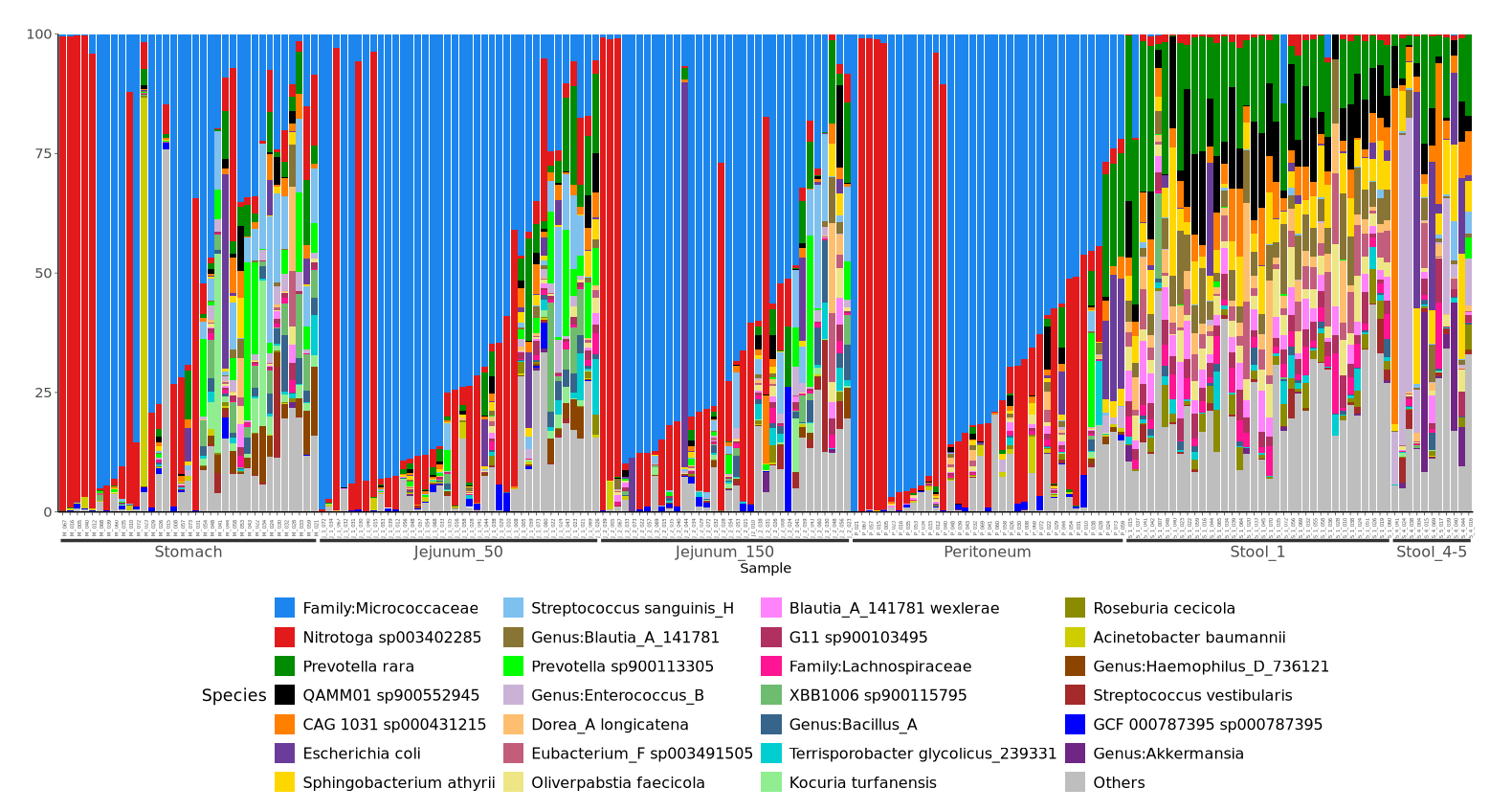

Supplement: Supplementary file 1 — Supplementary Material 1 [file 41598_2025_10931_MOESM1_ESM.png]

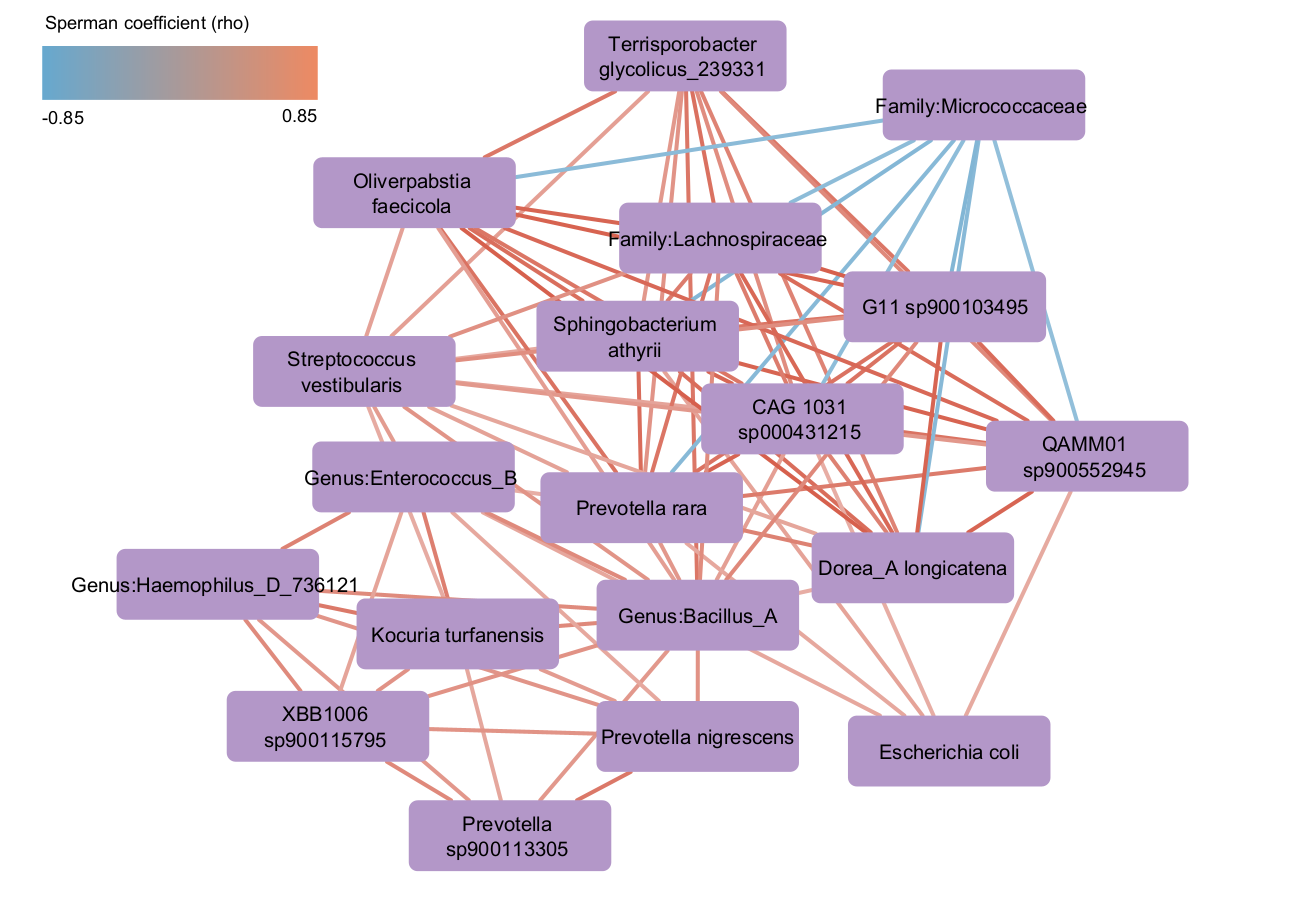

Supplement: Supplementary file 2 — Supplementary Material 2 [file 41598_2025_10931_MOESM2_ESM.png]

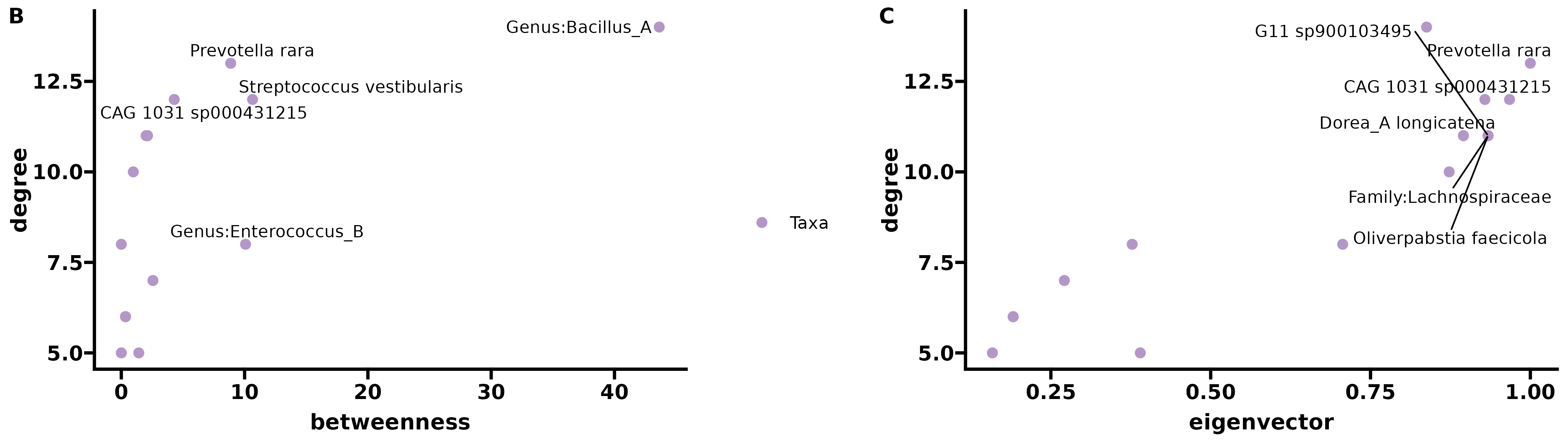

Supplement: Supplementary file 3 — Supplementary Material 3 [file 41598_2025_10931_MOESM3_ESM.png]

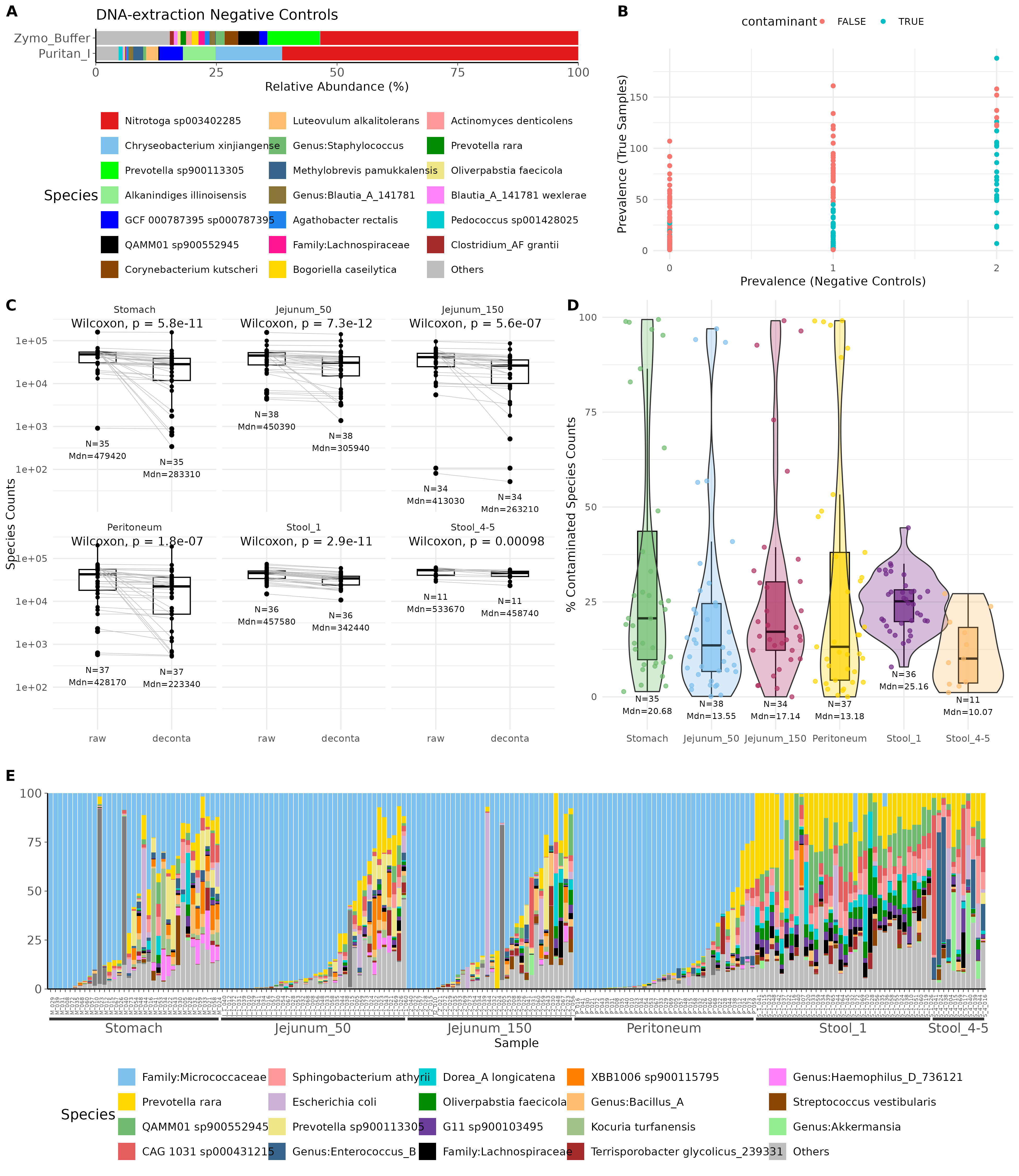

Supplement: Supplementary file 4 — Supplementary Material 4 [file 41598_2025_10931_MOESM4_ESM.png]
