# Supplementary material for: Examining spatial microbiome variations across gastrointestinal tract regions in obesity
Source: Sci Rep. 2025 Jul 14;15:25423. doi: 10.1038/s41598-025-10931-0 (PMC12260093; doi:10.1038/s41598-025-10931-0)
Supplement: Supplementary file 5 — Supplementary Material 5 [file 41598_2025_10931_MOESM5_ESM.pdf]

Characteristic	N	**Overall**, N = 191	Location group						q-value[1]
			**Jejunum 150**, N = 34	**Jejunum 50**, N = 38	**Peritoneum**, N = 37	**Stomach**, N = 35	**Stool 1**, N = 36	**Stool 4-5**, N = 11	
**Age**, Mean ± SD	190	47 ± 11	48 ± 11	48 ± 11	47 ± 11	47 ± 11	47 ± 12	48 ± 12	>0.99
**BMI**, Mean ± SD	191	48 ± 8	48 ± 9	48 ± 8	48 ± 8	49 ± 8	48 ± 6	46 ± 5	>0.99
**DNA [ng]**, Mean ± SD	191	2,257 ± 2,041	2,117 ± 1,172	1,957 ± 1,244	1,339 ± 871	1,640 ± 1,962	3,808 ± 2,890	3,704 ± 2,831	<0.001
**CRP value at inclusion mg-L**, Mean ± SD	187	9 ± 8	9 ± 8	9 ± 8	9 ± 8	9 ± 8	9 ± 6	6 ± 6	>0.99
**IL-6 at inclusion pg-ml**, Mean ± SD	182	5.7 ± 3.5	5.6 ± 3.6	5.5 ± 3.4	5.4 ± 3.4	5.6 ± 3.5	6.0 ± 3.4	6.3 ± 5.4	>0.99
**HbA1c perc**, Mean ± SD	188	6.14 ± 1.31	6.18 ± 1.18	6.25 ± 1.42	6.22 ± 1.43	6.29 ± 1.44	5.88 ± 1.17	5.65 ± 0.81	0.91
**Gender**, n (%)	191								>0.99
Female		138 (72%)	25 (74%)	28 (74%)	27 (73%)	25 (71%)	25 (69%)	8 (73%)	
Male		53 (28%)	9 (26%)	10 (26%)	10 (27%)	10 (29%)	11 (31%)	3 (27%)	
**Smoker**, n (%)	191								>0.99
Yes		59 (31%)	11 (32%)	12 (32%)	12 (32%)	11 (31%)	11 (31%)	2 (18%)	
No		132 (69%)	23 (68%)	26 (68%)	25 (68%)	24 (69%)	25 (69%)	9 (82%)	
**Fatty liver**, n (%)	191								>0.99
Yes		128 (67%)	22 (65%)	25 (66%)	24 (65%)	24 (69%)	26 (72%)	7 (64%)	
No		63 (33%)	12 (35%)	13 (34%)	13 (35%)	11 (31%)	10 (28%)	4 (36%)	
**Diabetes**, n (%)	191								>0.99
Yes		102 (53%)	21 (62%)	22 (58%)	21 (57%)	19 (54%)	14 (39%)	5 (45%)	
No		89 (47%)	13 (38%)	16 (42%)	16 (43%)	16 (46%)	22 (61%)	6 (55%)	

[1] False discovery rate correction for multiple testing
