# Supplementary material for: Examining spatial microbiome variations across gastrointestinal tract regions in obesity
Source: Sci Rep. 2025 Jul 14;15:25423. doi: 10.1038/s41598-025-10931-0 (PMC12260093; doi:10.1038/s41598-025-10931-0)
Supplement: Supplementary file 9 — Supplementary Material 9 [file 41598_2025_10931_MOESM9_ESM.docx]

**Supplementary Figures and Tables**

Supplementary Figure 1. Microbial profile displaying the relative abundance of 191 samples before decontamination, ordered by sampling locations. Species labels are shown only for the most abundant taxa, collectively representing 90% of the total abundance. Low-abundance taxa are grouped under the ‘Others’ category.

Supplementary Figure 2. A) Relative abundance of negative control samples: *Puritan_I*, containing the storage medium of the swabs used, and *Zymo_Buffer*, representing nucleic acid extraction using only the buffers provided by the DNA extraction kit. Species labels are shown only for the most abundant taxa, collectively representing 90% of the total abundance. Low-abundance taxa are grouped under the ‘Others’ category. B) Decontamination analysis results, displaying the prevalence each microbe (classified as contaminant or non-contaminant) in the true samples (Y-axis) and in the negative controls (X-axis). C) Species counts before and after the decontamination analysis, by sampling location. D) Fraction of species counts classified as contaminated, by sampling locations. E) Microbial profile displaying the relative abundance of the final set of 172 samples, after decontamination and ordered by sampling locations. Species labels are shown only for the most abundant taxa, collectively representing 90% of the total abundance. Low-abundance taxa are grouped under the ‘Others’ category. Abbreviations: N=number of samples; Mdn=median.

Supplementary Figure 3. A) Network displaying Spearman correlations (edges) on cumulative sum scaled species counts between the 20 most abundant bacterial species (nodes). Only correlations with a p-value < 0.05 and rho>0.4 are shown. Edges are coloured according to the correlation coefficient value. B) For each species node, the betweenness network centrality measure is displayed according to the node’s degree. Labels are only shown for the five species with the highest betweenness values. C) For each species node, the eigenvector network centrality measure is displayed according to the node’s degree. Labels are only shown for the six species with the highest betweenness values.

Supplementary Table 1. Samples sequenced for the study. Each sample is associated with its correspondent patient’s metadata.

Supplementary Table 2. Decontamination analysis results per species, displaying the frequency and prevalence of each contaminant and non-contaminant microbe. Abbreviations: freq=frequency; prev=prevalence; p. freq=p-value frequency method; p.prevalance=p-value prevalence method; p=p-value combined method.

Supplementary Table 3. Decontamination analysis results per sample

Supplementary Table 4. Spearman correlations on cumulative sum scaled species counts between the 20 most abundant bacterial species. Only correlations with a p-value < 0.05 and rho>0.4 are included. Abbreviations: cor=spearman’s rho correlation coefficient; p=p-value; q=p-adjusted value.
